# Enhancing Road Safety with AI-Powered System for Effective Detection and Localization of Emergency Vehicles by Sound

**DOI:** 10.3390/s25030793

**Published:** 2025-01-28

**Authors:** Lucas Banchero, Francisco Vacalebri-Lloret, Jose M. Mossi, Jose J. Lopez

**Affiliations:** Institute of Telecommunications and Multimedia Applications, Universitat Politecnica de Valencia, 46022 Valencia, Spain; lbanmar@upv.edu.es (L.B.); fvacllo@iteam.upv.es (F.V.-L.); jmmossi@dcom.upv.es (J.M.M.)

**Keywords:** sound detection, acoustic localization, emergency sounds, road safety, advanced driver assistance systems, transformers, ResNet, real-time systems, artificial intelligence, vehicles

## Abstract

This work presents the design and implementation of an emergency sound detection and localization system, specifically for sirens and horns, aimed at enhancing road safety in automotive environments. The system integrates specialized hardware and advanced artificial intelligence algorithms to function effectively in complex acoustic conditions, such as urban traffic and environmental noise. It introduces an aerodynamic structure designed to mitigate wind noise and vibrations in microphones, ensuring high-quality audio capture. In terms of analysis through artificial intelligence, the system utilizes transformer-based architecture and convolutional neural networks (such as residual networks and U-NET) to detect, localize, clean, and analyze nearby sounds. Additionally, it operates in real-time through sliding windows, providing the driver with accurate visual information about the direction, proximity, and trajectory of the emergency sound. Experimental results demonstrate high accuracy in both controlled and real-world conditions, with a detection accuracy of 98.86% for simulated data and 97.5% for real-world measurements, and localization with an average error of 5.12° in simulations and 10.30° in real-world measurements. These results highlight the effectiveness of the proposed approach for integration into driver assistance systems and its potential to improve road safety.

## 1. Introduction

The detection and localization of sound through artificial intelligence (AI) is gaining momentum due to advancements in deep neural networks and signal processing. Traditionally, audio processing has been a fundamental discipline, but it has evolved independently from AI, which has been more focused on fields such as vision [1,2,3] and natural language processing [4,5]. However, in the past decade, it has been demonstrated that audio is also a key area where AI’s capabilities can be leveraged to create high-impact systems. Identifying and localizing specific sounds, such as alarms and emergency sirens, presents a technical challenge that combines acoustics, pattern detection, and spatial inference. Its practical applications in real-world environments are still in the early stages.

Currently, numerous studies have developed models that detect [6] and localize [7] various sound sources in controlled laboratory environments and/or simulations. However, despite these advances, few of these systems have been able to move beyond the research domain to offer practical and accessible solutions for end users. This is primarily due to feasibility issues [8,9,10,11,12,13]: in real-world scenarios, complexity increases significantly due to environmental noise [14], variability in acoustic conditions [15], and hardware limitations [16], which reduce the robustness and accuracy of these systems. This work addresses these challenges and aims to bring sound detection and localization technology from the laboratory to real-world applications, particularly in the automotive sector.

The automotive context is particularly relevant and challenging for emergency sound detection systems [17]. When a driver hears a siren on the road, their immediate instinct is to identify its direction and location to respond appropriately, which temporarily diverts their attention from driving. This intuitive process, while effective, poses a significant risk, as any second spent searching for the sound’s origin is a moment of inattention. In a road environment where split seconds can determine the safety of the vehicle and its occupants, having an assistance system that quickly and accurately detects and localizes the emergency sound source can significantly reduce the risk of accidents [17].

The state-of-the-art in siren detection and localization has been approached in various ways across multiple patents and publications, highlighting significant differences in methodologies. For instance, Magna Electronics Inc. [18] proposes a system using cameras and microphones to detect siren sounds, processing both visual and acoustic data to determine the sound’s direction and confirm it originates from an emergency vehicle. This system exclusively employs mathematical techniques and algorithms without incorporating artificial intelligence. In contrast, Zoox Inc. [19] explores the use of a growing number of acoustic sensors to determine the Direction of Arrival (DoA) of the sound, aiming to identify and localize emergency sounds based on acoustic data, incorporating machine learning models for improved accuracy. However, this approach is oriented toward autonomous vehicles, not Advanced Driver Assistance Systems (ADAS).

The state of the art in onboard emergency vehicle detection and localization is not extensive, with relatively few studies addressing this critical area. Among the existing works, ref. [20] focuses on leveraging convolutional neural networks and gammatone filter banks to enhance the detection and localization of sirens and horns in noisy urban environments, achieving robust performance in scenarios with low signal-to-noise ratios. However, it does not address hardware adaptations for wind noise mitigation, which is crucial in high velocities. Additionally, the study relies on a two-microphone setup, which may lead to inconsistencies in localization, especially when comparing sounds in front of and behind the microphones. Furthermore, the training was conducted exclusively with simulated data, which can lead to falsely high accuracy values, as the behavior of simulated sounds does not fully replicate the complexities of real-world environments, such as occlusions caused by vehicles and their impact on the sound propagation and detection process. On the other hand, ref. [21] is limited to static sound localization without considering the movement of a siren in space, which is crucial for accurate localization. Additionally, none of these works address the issue of protecting microphones from wind, a critical factor in real-world scenarios. These limitations highlight the need for more advanced and precise solutions, like the one we propose.

Another critical issue is that, within the context of sirens in traffic environments, the majority of publications focus primarily on detection. In many cases, the discussion is limited to determining whether the siren is approaching or receding without advancing to real-time localization of the sound event [22,23,24,25]. This significant gap in the literature underscores the challenges of accurately identifying and tracking emergency sound sources in dynamic and noisy traffic scenarios, further emphasizing the need for systems capable of providing real-time localization to improve situational awareness and safety.

Furthermore, another significant challenge arises from the heavy reliance on simulated data across many studies in emergency sound localization [26,27]. While simulations provide an effective framework for controlled testing, they often fail to replicate real-world complexities, such as acoustic reflections, environmental noise, and occlusions caused by obstacles. This disconnect creates a gap between theoretical findings and practical applicability. In [28], an effort was made to address this by adapting models from [29,30] to predict not only the presence of emergency sounds but also their direction and distance. The commented models in [28] were evaluated using real-world data, revealing substantial localization errors, with angular deviations exceeding 35°. Moreover, these errors increased with the distance of the sound source, further emphasizing the limitations of models trained exclusively on simulated data when applied to the unpredictable conditions of real-world environments.

Addressing the limitations and gaps identified in the state-of-the-art, this study presents the development of an accessible and practical system for real-world applications, particularly within the automotive environment, and its integration with Advanced Driver Assistance Systems (ADAS) [31]. The system is designed to accurately identify and localize emergency sounds, providing the driver with a clear visual reference of the sound’s direction and proximity. The system’s ease of use and effectiveness are critical for ensuring quick decision-making without distracting the driver from the road.

In contrast to traditional scientific papers that typically follow the chronological progression of hypothesis formulation, validation, and iterative refinement, this work adopts a solution-first approach to enhance clarity and practical understanding. This structure is particularly beneficial for complex systems where multiple components interact synergistically. By presenting the complete architecture from the outset, it enables readers to grasp the interrelations of individual components before exploring the experimental validation. The challenge this study addresses lies in the difficulty of accurately detecting and localizing emergency vehicles in dynamic road environments, where traditional methods often fail due to fluctuating acoustic conditions and environmental noise. This research introduces a novel solution by integrating advanced microphone array configurations, adaptive acoustic devices, and artificial intelligence models to build a robust system that can reliably detect and localize emergency vehicles in real-time, even in complex scenarios. Through this integration, the study aims to overcome the limitations of previous systems, offering a real-time, scalable solution that significantly improves emergency response times. The design of the microphone array, the development of AI models for sound detection, and the extensive validation process together form a comprehensive framework that ensures both the system’s effectiveness and robustness in real-world conditions. Beyond its immediate technical contributions, this work also advances the understanding of acoustic signal processing in challenging environments, offering a practical application that enhances road safety and optimizes emergency response.

The paper is organized as follows. Section 2 presents the overall system architecture, establishing the framework within which all components operate. Section 3 details the microphone array configuration and acoustic adaptation devices, which form the critical foundation for high-quality signal acquisition. Section 4 introduces the artificial intelligence models that process these signals for sound detection and localization. Section 5 describes the comprehensive data collection and synthesis methodology that enabled both system development and validation. Finally, Section 6 presents the experimental validation and performance analysis, demonstrating how the integrated system achieves robust performance under real-world conditions.

## 2. Proposed System Architecture

This section presents the resulting system architecture that emerged from our extensive research and development process. The architecture’s effectiveness is comprehensively validated in Section 6, using the diverse dataset collected through the measurement campaign detailed in Section 5. The system’s design addresses the fundamental challenges of emergency sound detection in automotive environments through four interconnected subsystems: acoustic signal acquisition, noise reduction and enhancement, emergency sound detection, and sound source localization. The proposed system is based on a continuous process of listening, analyzing, and localizing emergency sounds, operating under a real-time analysis approach.

**Figure 1:**
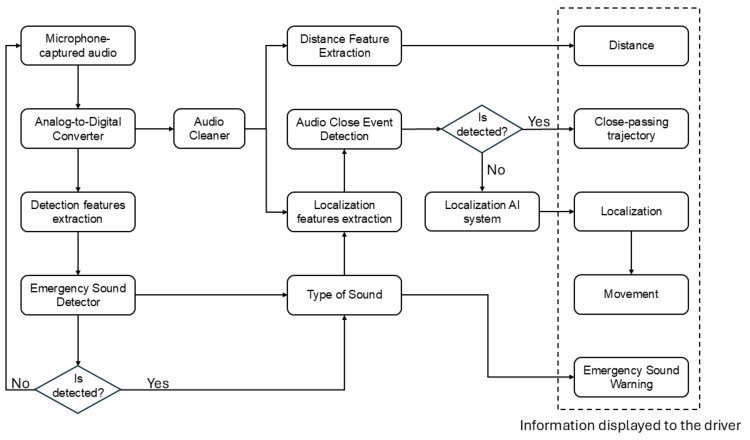
Block diagram of the proposed system.

Figure 1 presents the block diagram of the entire system without delving into the specific details of each component, which will be explained in the following sections. This modular approach is crucial because, although each component is effective on its own, it is only through their interaction that the system achieves its maximum performance. The ability to continuously analyze data, clean emergency signals, and localize their spatial origin is a complex task that requires precise synchronization of all blocks.

Firstly, the system listens to the environment at one-second intervals, accumulating data in four-second blocks. This is performed using a process known as a sliding window in which the system listens for one second, and once four seconds are reached, it analyzes the data. After processing the first four-second block, the system discards the first second of the buffer and adds a new one, continuously repeating this cycle. This approach ensures the system is constantly updated with the most recent sounds while managing the information efficiently.

The audio collected in each four-second window is sent to the first detection block, the Emergency Sound Detector, based on artificial intelligence. This block is responsible for identifying whether or not an emergency sound, such as a siren, is present within the analyzed audio segment. If no emergency sound is detected, the system simply moves on to the next window, repeating the listening and analysis process.

If, on the other hand, an emergency sound is detected, the system proceeds to the next block, the AI Cleaner. This component filters the sound by removing any background noise or irrelevant interference, extracting only the audio corresponding to the emergency sound. This cleaning process is performed using an artificial intelligence system trained to recognize and separate the emergency sound from other noises in the environment.

After the emergency sound has been cleaned, the audio moves to the next stage, the Locator. This block is crucial as it determines the position of the sound relative to the vehicle. Since the system operates with four-second segments, the locator has two analysis branches. The first branch detects whether the emergency sound has passed quickly around the vehicle, meaning it is a relatively nearby traffic sound. If so, the system detects the direction of the sound and determines the path it took. If, however, no rapid pass is detected, the system activates the second branch, which precisely calculates the exact location of the emergency sound, its distance, and whether it is approaching or moving away from the vehicle.

**Figure 2:**
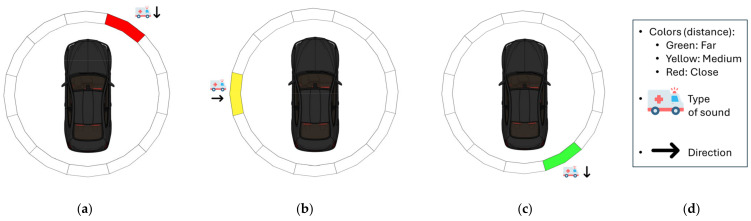
Examples of acoustic environment representation on the onboard computer: (**a**) siren approaching from a short distance at approximately 30°; (**b**) siren approaching from a medium distance at approximately 270°; (**c**) siren moving away from a long distance at approximately 150°; (**d**) color-coded proximity indicators with symbol explanations, including the type of sound and estimated movement direction.

Once all these processes are completed and the relevant information about the position and behavior of the sound has been obtained, the data are presented to the user in a clear and understandable manner. This presentation is performed through a simple interface designed to allow the driver to quickly interpret the information and make informed decisions without compromising attention to the road. As shown in Figure 2, the system displays the results visually, indicating the direction, proximity, and status of the emergency sound. Red, yellow, and green colors are used to reflect the proximity and status of the sound, where red indicates an immediate threat, yellow represents a moderate risk, and green signals a safe condition. This enables the driver to react efficiently and safely.

Each subsystem has been carefully optimized to complement the others, creating a robust solution that maintains high performance even in challenging acoustic environments. The success of this architecture relies heavily on the specialized microphone array configuration (detailed in Section 3) and the sophisticated AI models (presented in Section 4).

The importance of concatenating these systems is reflected in the continuous improvement of predictions and system accuracy, as shown in Section 6, enabling better adaptation to highly complex acoustic environments.

## 3. Microphone Array Configuration and Acoustic Adaptation

This section describes the final microphone array configuration and acoustic adaptation devices that form the cornerstone of our system’s performance. The arrangement we present represents the optimal configuration identified through extensive testing, specifically designed to provide high-quality inputs to the AI models described in Section 4. The configuration consists of strategically positioned weatherproof microphones integrated into the vehicle’s exterior, complemented by custom-designed aerodynamic shields and vibration isolation systems. This hardware foundation proved crucial for achieving the performance metrics presented in Section 6, particularly in challenging real-world conditions.

**Figure 3:**
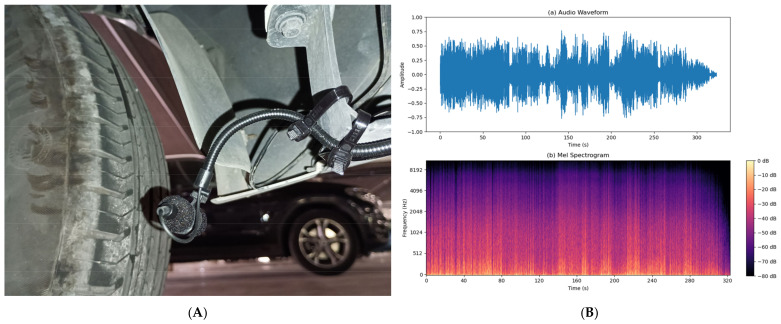
Initial noise measurement: (**A**) Microphone placement setup; (**B**) Measurement results.

The effectiveness of any detection AI-based system fundamentally depends on the quality of its input signals [32]. Wind and vibration noise contamination of audio signals represents one of the most challenging problems in sound capture systems for vehicles in motion. This phenomenon was identified during the initial tests conducted with the first setup for road sound recording (shown in Figure 3A), where it was observed that the airflow over the microphones generated a constant and intense environmental noise. After recording the signals captured under road conditions, a detailed analysis of the measurements was performed. These analyses revealed that wind introduces significant interference in the frequency spectrum, which is expected to complicate detection and drastically reduce the accuracy of localization systems. In particular, wind noise masks critical informational frequencies in emergency sounds and generates artifacts in the audio that may hinder the optimal functioning of AI algorithms designed to identify and localize emergency sounds in real-time, as can be seen in Figure 3B.

To address this challenge, in our previous work [33], a specific aerodynamic protective structure was designed for the system’s microphones, which significantly reduces the intake of wind noise and vibrations associated with driving. The system is designated as the Instrument for Capturing External Communication and Reliable Environmental Audio Monitoring (hereafter referred to as ICECREAM).

**Figure 4:**
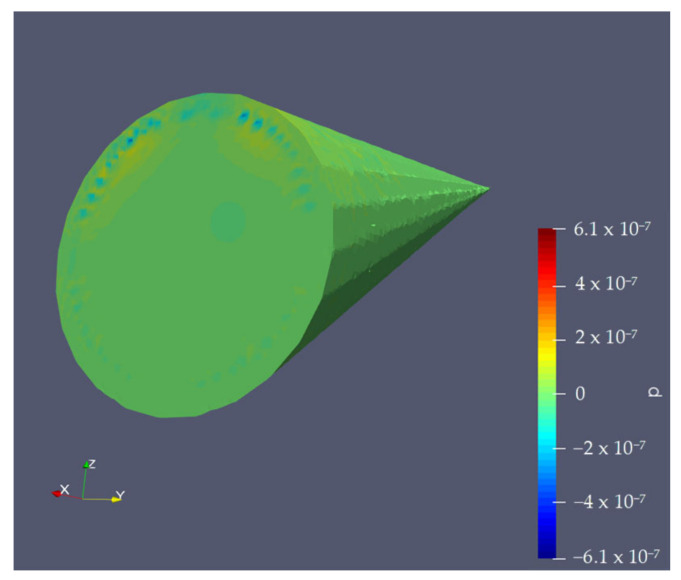
Aerodynamic simulation of pressure variation of the structure at 50 km/h.

The protective piece consists of a tapered cone-shaped casing with a flat base designed to reduce turbulence in the area near the microphone. The geometry of this casing is not arbitrary; it is optimized to deflect the airflow in the most aerodynamic way possible, thus minimizing the direct impact of the wind on the device’s diaphragm. This design reduces the noise generated when wind directly strikes the microphone, a common issue in audio capture setups in open or moving environments. In tests conducted on turbulence simulations in [33], shown in Figure 4, it has been verified that this structure deflects most of the airflow, allowing for cleaner sound capture.

Plastic was chosen as the material for the outer casing due to its water resistance, durability, light weight, and ease of 3D printing. The opening at the base is strategically placed and surrounded by a raised edge that acts as a safeguard, as studies in [33] have shown that the areas near the outer edges are where the highest wind turbulence occurs.

Inside this protective structure, an acoustic absorbent material has been included. This acoustic absorbent not only helps dampen mechanical vibrations that may transfer from the vehicle to the microphone through the casing but also reduces internal resonances that could amplify noise [34]. The choice of this material was made following a series of experimental tests, which showed that the absorbent foam was able to significantly attenuate noise in the 500 Hz and 2500 Hz frequency bands, where wind and vibrations typically have a stronger impact [10,35].

To further reduce vibrations, the microphone is “floating” inside the protective casing, suspended within the absorbent material that decouples it from the vehicle’s rigid structure. This setup prevents the transmission of vibrations from the casing, which is physically connected to the vehicle, to the microphone.

**Figure 5:**
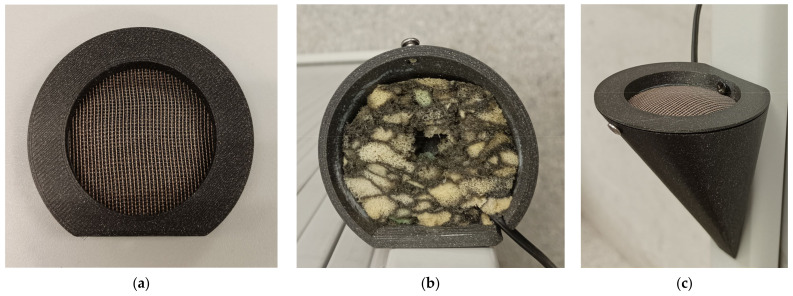
ICECREAM Design: (**a**) Cover with windproof filter; (**b**) Interior of the structure filled with absorbent material; (**c**) Complete piece.

**Figure 6:**
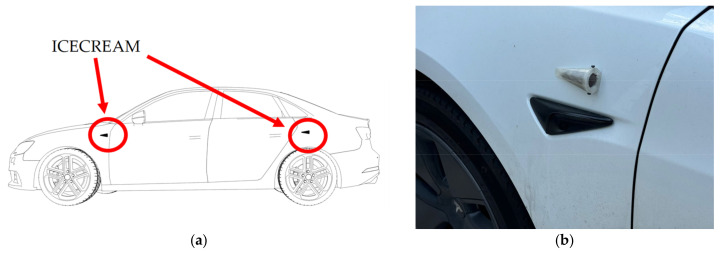
Real design of ICECREAM: (**a**) sketch of the lateral placement position of ICECREAM on the vehicle; (**b**) ICECREAM mounted on the vehicle.

To complement the wind protection, the casing’s opening features a windproof filter designed to block the passage of residual air that may enter through the hole. This filter acts as an additional barrier, reducing the amount of wind that could directly contact the microphone diaphragm, thus preventing the residual airflow from causing interference in the captured signal. The complete piece and its details are shown in Figure 5, while Figure 6 illustrates the positions in the vehicle (a) and their corresponding close-up view (b).

## 4. Artificial Intelligence Model Architecture

Building upon the high-quality acoustic inputs provided by the hardware infrastructure described in Section 3, this section presents our comprehensive AI framework for emergency sound processing. While the complete validation using real-world data is presented in Section 6, the architecture detailed here represents the culmination of extensive model exploration and optimization. The development process involved iterative testing with both simulated and real-world data (as detailed in Section 5), leading to a system that employs a combination of transformer-based models and convolutional neural networks (CNNs), leveraging their respective strengths in temporal signal processing and spatial feature extraction. This synergistic combination was specifically designed to leverage the strengths of each approach while compensating for their individual limitations in automotive acoustic environments.

### 4.1. AI-Based Detection System

As commented in Section 2, the proposed system operates with four-second audio blocks processed through a sliding window approach. This allows the system to continuously analyze the most recent sound data, maintaining a balance between constant information updates and efficient processing management. The system’s initial block, the Emergency Sound Detector, is responsible for analyzing these audio segments to identify the presence of relevant acoustic events, such as sirens and horns, which are considered the primary emergency sounds on the road. This detector serves as a trigger for the subsequent modules, making its accuracy and robustness crucial.

The detector must be highly precise to avoid false alarms and ensure that the subsequent modules are only activated when emergency sounds are actually detected. At this stage, the system does not yet benefit from the cleaning module, so the model must operate effectively in noisy sound environments typical of roads and urban settings. This means that the detector must be capable of distinguishing between emergency sounds and background noise, such as that generated by traffic, wind, or vehicle vibrations.

To address this challenge, Mel spectrograms have been chosen as the primary descriptor. This type of spectral representation allows for the preservation of both temporal and frequency information from the audio, which is essential for capturing the distinctive characteristics of emergency sounds. Furthermore, the Mel spectrogram provides higher resolution in the lower frequencies, a range where much of the ambient noise typically resides. This approach is particularly relevant as it enables the model to extract specific acoustic patterns from sirens and horns, even in the presence of significant noise.

**Figure 7:**
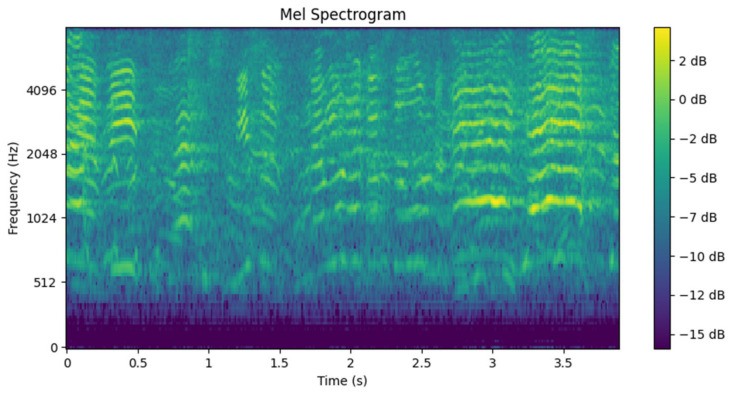
Mel spectrogram of a siren sound sample.

The Mel spectrogram is generated using 128 Mel filters, a 32 ms analysis window (defined by the size of the Fourier transform, nfft), and a hop length of 10 ms, ensuring an appropriate temporal resolution to capture the dynamics of emergency sounds. These configurations balance both temporal and frequency resolution, allowing for a detailed representation of the acoustic content within the analyzed segments. As a result, a spectrogram is obtained, such as the one shown in Figure 7, which serves as input to the detection model.

Once the input parameter for the event detector was determined, we focused on selecting an AI model capable of accurately detecting relevant sounds with minimal error. Although several AI architectures were tested for this task, the model based on transformers [36], specifically the Audio Spectrogram Transformer (AST) [37], provided the best performance in terms of accuracy and generalization. The tested architectures included convolutional neural networks (CNNs) such as ResNet and VGG, as well as custom CNNs designed specifically for this system. Additionally, recurrent neural networks (RNNs) like LSTM were explored, both individually and in hybrid architectures combining CNNs for spectrogram pattern analysis with subsequent LSTM layers for temporal feature extraction. Despite the promising results of these models, the transformer-based architecture demonstrated superior accuracy and generalization, making it the most suitable choice for this task. This is due to its ability to capture complex relationships in audio spectrogram representations, as demonstrated in [37]. These architectures were specifically adapted for emergency sound detection tasks, following a similar approach to the one described in [38]. Specifically, the final layers of the AST model were modified to specialize in classifying three categories: siren, horn, and other sounds or noise. This approach effectively filters out irrelevant signals, ensuring that only critical events trigger subsequent modules. To achieve this, the weights of the Multihead Attention blocks [36] were frozen, and the classification neurons were modified by adding a 768-neuron layer with a Parametric Rectified Linear Unit (PReLU) activation, followed by a layer of 3 neurons with a Softmax activation, classified as Horn, Siren, and None.

The hypothesis behind this change is that the system will be able to distinguish between the three defined types of sounds, accurately classifying sirens, horns, and other irrelevant noises. When one of the emergency sounds is detected, the system will alert the driver, providing the necessary information to respond quickly and appropriately. In Section 6, the results and validations of this detection approach will be presented, demonstrating its accuracy and reliability in classifying and responding to emergency sounds in real-time.

### 4.2. AI-Based Cleaning System

As described in the process referenced in Section 2, the next step after emergency sound detection is the cleaning of the audio (noise removal) for subsequent analysis. This process complements the anti-wind hardware system described in Section 3, working together to eliminate unwanted noises that may contaminate the signal and affect the system’s accuracy in later stages.

As developed in [33], the system uses the spectrogram of the signal as the input feature, utilizing the Short-Time Fourier Transform (STFT). First, the magnitude and phase information of the spectrogram are separated. Since the main objective is to reduce frequencies corresponding to noise and irrelevant features while preserving emergency sounds, the processing is performed only on the magnitude, keeping the phase intact. This decision is critical, as preserving the phase ensures that the temporal structure of the signal remains unaltered, which is essential for accurate audio reconstruction. Once the magnitude has been cleaned, the phase is re-integrated, and the inverse STFT is applied to reconstruct the audio from the cleaned spectrogram.

For the spectrogram extraction process, a nfft value of 54 ms and a hop_length of 3 ms have been used. This choice of parameters was made for two reasons. First, to ensure that the spectrograms have a square, regular shape, making it easier for the AI network to process, as will be discussed later. Second, the large nfft value combined with the small hop_length ensures that the spectrogram has a very high resolution, capturing all possible details of frequency variation over time, which helps avoid losing any information during the cleaning process.

This is how the ICECREAM system completes the cleaning of audio that has not yet been fully processed, particularly in the lower frequencies, a topic discussed in more detail in Section 6. Since 2D matrices are being analyzed, the most common approach is to use convolutional neural networks (CNNs) capable of processing and extracting relevant features from the spectrograms, as these networks are specifically designed to work with image-like data.

**Figure 8:**
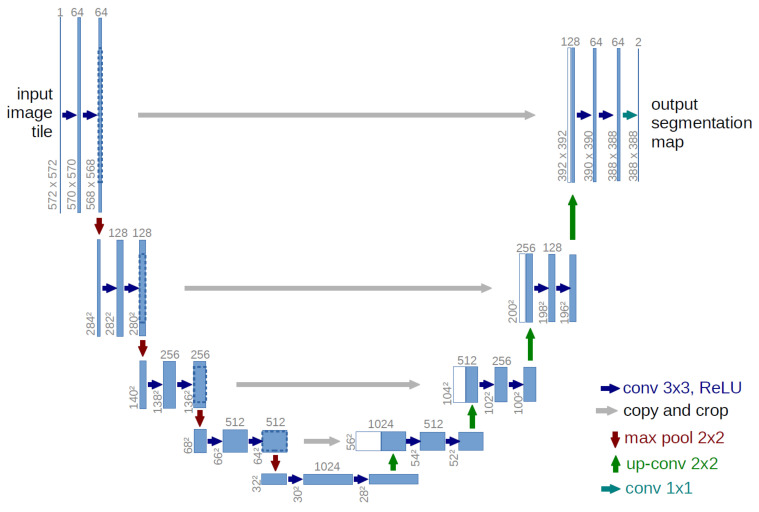
Example of U-Net Structure [39].

After testing several architectures, the U-Net [31,32,33,34] with skip connections was selected as it provided the best results in terms of signal preservation and noise reduction. As illustrated in Figure 8, this architecture proved particularly effective for the audio cleaning task due to its ability to learn efficiently during the “bottleneck” phase of the network training, allowing it to extract and expand the most relevant features of the spectrogram. Skip connections are especially valuable as they enable features learned in the early layers of the model to be effectively passed on to the deeper layers, improving the preservation and reconstruction of the key parts of the spectrogram. This feature is essential for maintaining audio quality after the cleaning process. Both the input and output of the model have the same dimensions as the spectrogram, which facilitates reconstruction without loss of information.

After a practical and experimental training study with the data obtained in Section 5, it was concluded that U-Net networks can introduce certain homogeneous noise artifacts in the spectrogram output. Therefore, to address this issue, a Wiener filter [40] was applied before reintegrating the phase. This filter is essential for removing any white noise that may have been introduced during the cleaning process, ensuring that the final spectrogram is free of interference and ready to be processed by the subsequent stages of the localization system. In this way, the network not only effectively cleans the signal but also preserves key information to ensure that emergency sounds, such as sirens and horns, are optimally preserved for analysis and localization in the system. In Section 6, the results and validations of this cleaning approach will be presented, demonstrating its effectiveness in enhancing audio quality.

### 4.3. AI-Based Close-Passing Trajectory System

The following block of the system, as specified in Section 2, is responsible for the localization of emergency sounds when they are produced by sources moving near the vehicle. Due to the analysis approach using a sliding window of 1 s, where 4-s audio blocks are analyzed, situations may arise where a siren passes by the vehicle during the analysis period. This causes significant variations in the phase and amplitude signals received by the four microphones of the vehicle within that 1-s window. Due to this dynamic behavior, the system is divided into two localization branches: one for static sounds and another for those in motion near the vehicle.

This system has two main functions: one that detects whether there is a close-passing trajectory of the emergency sound relative to the vehicle and another that determines the trajectory of the emergency sound if it is in proximity. If it is detected that the sound is not passing near, the system acts as a trigger for the next block, which is responsible for detecting static sounds at a greater distance, as detailed in Section 4.4.

For the detection of a close pass, the temporal information arriving at the four microphones is essential. To achieve this, the Generalized Cross-Correlation with Phase Transform (GCC-PHAT) parameter [41,42] is used between pairs of microphones. This parameter allows the calculation of the phase shift between different microphone pairs and provides information on the relative localization of the sound. The GCC-PHAT is computed for each pair of spectrograms obtained during the analysis of the 4-s segments from each microphone, enabling precise extraction of the temporal information from each pair.

**Figure 9:**
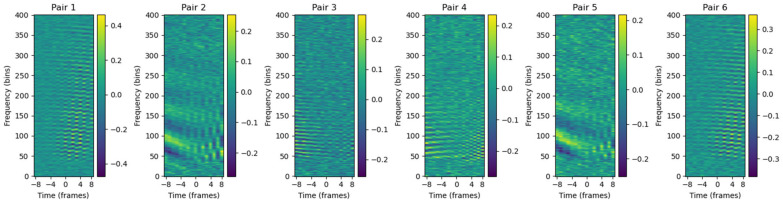
Different Examples of GCC-PHAT Between Different Microphone Pairs.

The spectrogram is generated with a 50 ms window duration (nfft) and a hop_length of 1 ms, ensuring a high temporal resolution essential for the analysis of phase shifts between microphones. Since this is a cross-correlation function applied to a spectrogram, the result is a 2D matrix that is twice the size of the spectrogram length, where the central point represents a zero-phase shift between the microphones. Given that the system is aware of the microphone locations, the maximum possible phase shift between them can be calculated. This allows for cropping the GCC-PHAT signal for each microphone pair, limiting the information to what is relevant for precise localization, leaving information as shown in Figure 9.

With four microphones, there are six possible pairs without repetition, and each of these pairs is calculated independently. Afterward, the GCC-PHAT matrices are stacked along the third dimension, similar to how an image has three dimensions (height, width, RGB). In this case, the input features are organized with three dimensions corresponding to the dimensions of the spectrogram and the six possible microphone pairs.

Once the GCC-PHAT features are obtained, they are fed into an artificial intelligence model based on residual convolutional neural networks (ResNet) [43], specifically the ResNet-18 version [43]. ResNet networks are widely known for their ability to learn deep representations of complex data, thanks to their skip connection structure. These connections allow relevant features to be efficiently transmitted through the network without losing information, which is essential for the processing of precise acoustic signals, such as those handled in this system. Additionally, ResNet-18 does not use max pooling layers between the convolutional layers, a common practice in this type of analysis, allowing the system to work with smaller matrices, like those generated by the GCC-PHAT calculation, without losing important details during the learning process.

**Figure 10:**
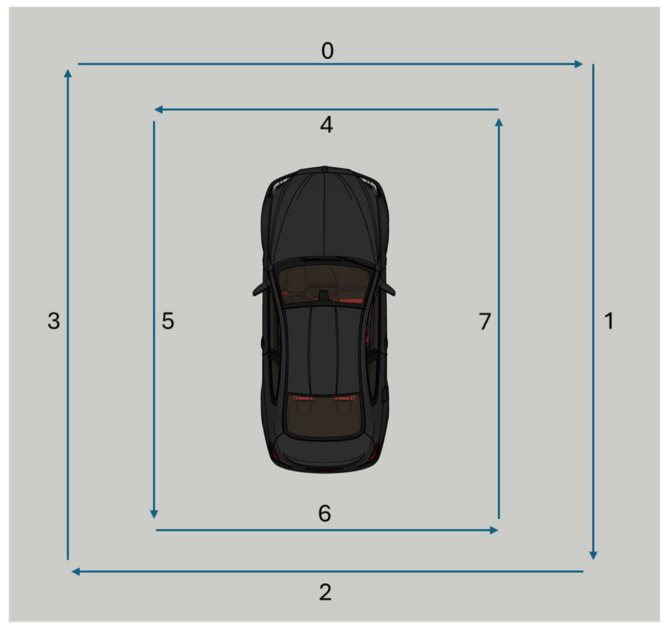
Different Types of Trajectories in Nearby Environments.

At the output of the ResNet, classification layers are added, consisting of a fully connected (fc) layer followed by a ReLU activation, a dropout layer to prevent overfitting, and a final classification layer with softmax to obtain the probability for each class. In this case, this first branch is configured to classify among 8 possible classes, corresponding to the 8 different possible close-passing sound trajectories, labeled from 0 to 7, as shown in Figure 10. These classifications are based on the temporal and spatial data collected by microphones.

Although several architectures were tested, such as traditional convolutional networks and more complex models with transformers, it was determined that ResNet-18 provided an optimal balance between accuracy and computational efficiency for this specific task. The residual connections help preserve the features learned in the initial layers, while the later layers add greater abstraction capacity. This was crucial for maintaining the quality of the information processed in the GCC-PHAT spectrograms, maximizing the system’s performance.

Once the model for detecting nearby events was trained, the next step was to modify the network to add a new branch, which would operate as the detector for nearby events in parallel with the original classification branch.

The new branch was designed to classify the presence or absence of a nearby event to the vehicle. It was constructed with a linear layer of 512 neurons, followed by a ReLU activation and a dropout layer to prevent overfitting. Then, another linear layer was added to adjust the output to two neurons with a softmax activation. The first neuron is activated if there is no nearby event, while the second neuron indicates that a nearby event is present. This design allows the system to simultaneously detect and classify nearby events. This structure complements the output of the original classification branch to provide an accurate localization of the events.

The model benefits from transfer learning, as the pre-trained ResNet has learned to identify different types of events, such as sirens and other emergency sounds. The process followed involves fine-tuning the modified model by freezing the weights of the previously trained ResNet-18 and only training the newly added branch with new data. These data not only include audio from different types of pass-by sounds but also audio with static sounds.

**Figure 11:**
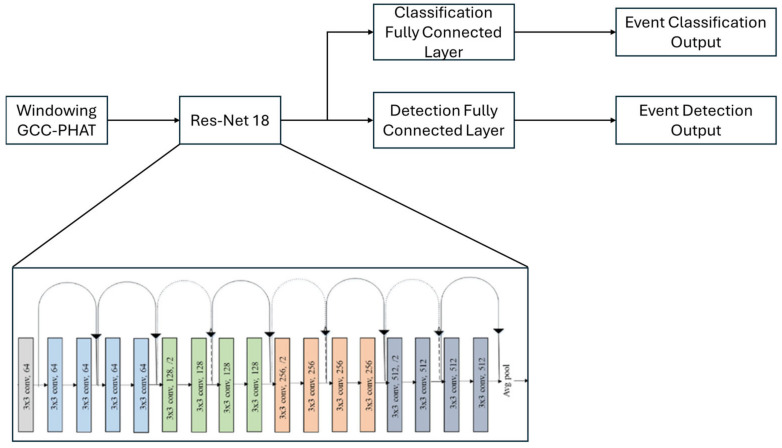
Schematic of the Architecture Modification for the Close-Passing Trajectory System Based on AI Model.

With the addition of the new branch, the model is structured as shown in Figure 11, allowing it to distinguish whether an emergency sound is occurring near the vehicle, which triggers the classification of the nearby sound’s trajectory. If no nearby event is detected, the classification output is discarded, and the system proceeds to analyze events at a greater distance. This modification of the model enables effective discrimination between fast-moving trajectory events or static sounds, optimizing the localization processing. In Section 6, the results and validations of this model will be discussed, demonstrating its capability to accurately classify and localize sound sources based on proximity and movement.

### 4.4. Static Localization System Based on AI

After completing the analysis of the close-passing trajectory in the previous section and determining that no such movement exists, the trigger activates the static sound localization process, as explained in Section 4.2. This locator is responsible for identifying the spatial location of sounds, using the Generalized Cross-Correlation with Phase Transform (GCC-PHAT) as an input parameter, which was also used in the previous stage to analyze the time delays between the microphones. By utilizing the same parameter for two distinct software blocks, computational savings are achieved in terms of processing time and overall system cost.

Since no movement event is detected, as determined by the previous block, this part of the system focuses on localizing static sounds using the temporal and spatial information from the microphones over time, understanding that these sounds change very slowly and can be effectively analyzed within a 1-s window, as the information does not vary significantly during this period.

Although different architectures were tested for this task, it was determined that the ResNet-18 network was once again the most suitable for spatial localization problems. Among the evaluated alternatives, ResNet-18 provided the best balance between accuracy and computational efficiency. Furthermore, the residual connections in this network promote the efficient transmission of information across layers, which is crucial for preserving signal quality in the context of precise sound localization.

However, on this occasion, although the proposed model is again based on a ResNet-18 network, a modified version is implemented, complemented with two separate branches. Each branch leverages the information learned by the network’s convolutional layers to estimate a trigonometric component of the sound’s direction of arrival: one branch predicts the cosine value of the angle, while the other predicts the sine value.

**Figure 12:**
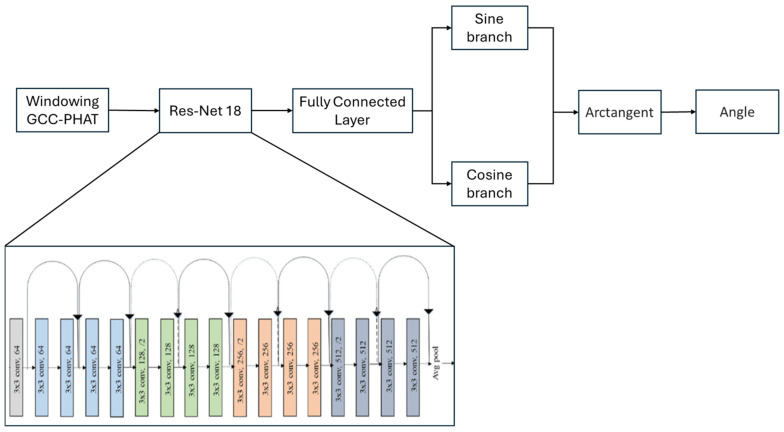
Schematic of the Architecture Modification for the Static Localization System Based on AI Model.

The goal is to predict the angle of arrival of the sound based on the features extracted from the microphones. The process begins with the ResNet-18 network, which extracts the relevant features from the spectrogram. These features are then passed to the two parallel branches, where each branch generates an output related to a trigonometric component of the angle: cosine and sine. Each branch consists of a fully connected layer with 512 neurons, followed by a PReLU activation, and then a single neuron layer with a Tanh activation function to map the output to continuous values in the range of −1 to 1, as the sine and cosine values of angles lie within this range, as shown in Figure 12.

Once the outputs from the two branches are obtained, corresponding to the cosine and sine values of the angle, the information is combined using the arctangent function, which calculates the angle from these two trigonometric components. The use of arctangent allows for an angle value in the range of 0° to 359°, corresponding to the direction of arrival of the sound.

The model is designed such that, after processing in the cosine and sine branches, the network outputs a single neuron that determines the sound’s angle of incidence. In this way, the model is capable of mapping the acoustic features extracted by ResNet-18 to a precise spatial angle, which is essential for the accurate localization of sounds in the space around the vehicle. In Section 6, the results and validations of this network will be presented, demonstrating its effectiveness and accuracy in determining the sound source’s position. The performance of the model will also be assessed through various metrics to ensure its robustness and reliability for practical applications.

### 4.5. Distance Estimation Algorithm

As shown in Figure 2, it is essential to provide the driver with clear and simplified information that allows them to quickly identify all the necessary details to take appropriate action. One of the most relevant variables in this context is the distance to the emergency vehicle, as knowing this will enable the driver to estimate the time available to make proper decisions. Therefore, it is proposed to classify the sounds into three categories: near range, medium range, and far range, visually represented by color codes (red, yellow, and green), as explained in Section 2.

In this case, artificial intelligence is not used, and instead, classical audio analysis algorithms are implemented, which are equally effective for this task. The problem is addressed with the understanding that determining distance through waveform amplitude is complex due to the inherent variations in the emission characteristics of different sirens. This makes it unfeasible to establish a universal threshold based on amplitude for all sirens.

However, there is physical evidence supporting the fact that at long distances, attenuation due to air absorption, as specified in ISO 9613-1:1993 [44], and interaction with surfaces such as asphalt [45] significantly affect high frequencies. Sirens emit a fundamental frequency range between 500 Hz and 1600 Hz [46], which, although relatively unaffected by distance, experiences more pronounced attenuation in its higher harmonics. Therefore, the analysis focuses on the relationships between the energies of the higher harmonics, where the attenuation effects are more significant.

**Figure 13:**
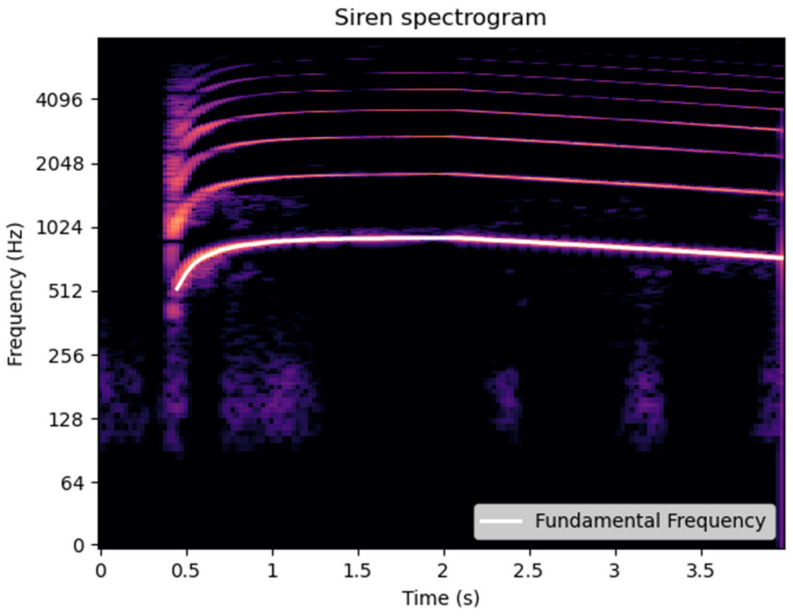
Fundamental Frequency of a Typical Case Extracted with the YIN Algorithm in a Spectrogram with a Siren Sample.

The proposed approach employs the YIN algorithm [47,48,49] to identify the fundamental frequency of the signal, leveraging prior knowledge about the nature of siren sounds. This algorithm, applied over a specific frequency range, allows for precise determination of the fundamental frequency, as shown in Figure 13. Since the signals are already cleaned after passing through the denoising block described in Section 4.2, the fundamental frequency detected by the algorithm is inherently that of the emergency sound. From this fundamental frequency, the corresponding harmonic frequencies are extrapolated, and the energy of each harmonic is then calculated.

The methodology avoids directly relying on the fundamental frequency due to its low attenuation with distance, as discussed previously. Instead, an exhaustive experimental analysis of the measurements in Section 5.4 revealed that the attenuation behavior of harmonics varies with distance. Specifically, higher harmonics experience less attenuation at shorter distances. To determine if a sound is within the “near” range, the energy ratio (E*_N_*) between the third harmonic and the sixth harmonic is computed, as represented by the following equation:

(1) $$E_{N}=\frac{E_{3}}{E_{6}},$$

where E_3_ and E_6_ represent the energy levels of the third and sixth harmonics, respectively, over time. When this ratio is small, it indicates that higher harmonics, such as the sixth, have retained significant energy, suggesting that the sound source is close to the vehicle.

To classify a sound as being in the “far” range, the energy ratio between the fundamental harmonic and the third harmonic is analyzed. At longer distances, the third harmonic undergoes greater attenuation due to air absorption and environmental interactions, as discussed earlier, leading to a progressive loss of its energy. This relationship is expressed as:

(2) $$E_{F}=\frac{E_{0}}{E_{3}},$$

where E_0_ corresponds to the energy of the fundamental harmonic, and E_3_ represents the third harmonic energy. A high ratio indicates that the third harmonic has lost significant energy, placing the sound source in the “far” range.

Based on the extensive experimental analysis from Section 5.4 and the validation results in Section 6.5, specific thresholds are established to classify the sound source into three distance ranges: near, medium, and far. This harmonic energy ratio-based approach provides a reliable method for estimating the proximity of emergency sounds, significantly enhancing the accuracy of the acoustic localization system.

## 5. Data Collection and Synthesis

The success of both the hardware configuration (Section 3) and the AI models (Section 4) critically depends on the quality and diversity of the training and validation data. This section outlines our comprehensive data acquisition and synthesis framework, designed to ensure robust system performance across a wide range of operational conditions. The methodology encompasses four complementary approaches, each addressing specific aspects of the system’s operational requirements: Real Environment Noise Capture, Indoor Simulation of Outdoor Environments, Synthetic and Semi-Synthetic Outdoor Environment Generation, and Motion and Real-world Environment Capture.

Section 5.1, Section 5.2 and Section 5.3 detail our approach to creating a robust training corpus through the strategic combination of real, semi-synthetic, and synthetic data. This hybrid approach enables us to cover a broader range of scenarios than would be possible with real data alone, while Section 5.4 focuses on the acquisition of validation data in actual operational conditions, providing the foundation for the performance analysis presented in Section 6.

### 5.1. Real Environment Noise Capture

**Figure 14:**
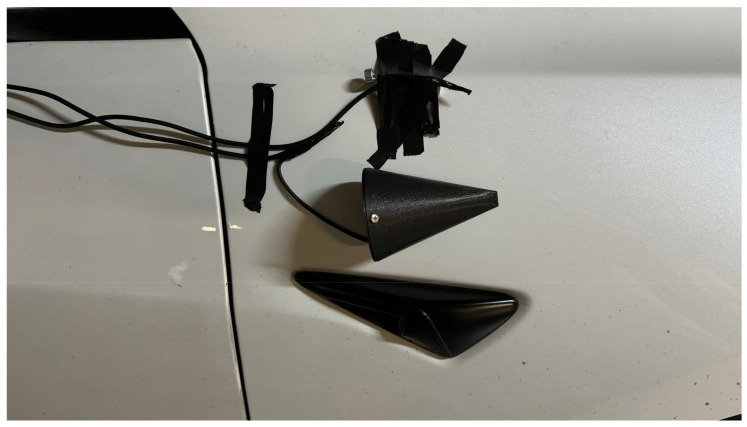
Microphone Setup Mounted on the Vehicle’s Body.

With the implementation of the ICECREAM system, data collection was carried out using two configurations simultaneously, as shown in Figure 14: an unprotected electret microphone and the same electret microphone with the ICECREAM housing. This process allowed for the evaluation of the effectiveness of the ICECREAM structure in protecting the microphones from external interference. Data collection was conducted by varying both the speed and the type of roads, covering speed ranges from 20 km/h in urban environments to 120 km/h on highways.

This data collection process was crucial for several reasons. First, it allowed for a comparison of the performance of different microphones in real-world conditions and evaluated the effectiveness of the ICECREAM structure in protecting the microphones from external interference. Additionally, data collection at various speeds and in different system configurations enabled the simulation of realistic scenarios, which were later used to train and validate the artificial intelligence algorithms. This simulation of real-world environments was essential for improving the accuracy of the models and ensuring that the results are applicable in real-world situations, thereby optimizing the system’s reliability in practice.

### 5.2. Indoor Simulation of Outdoor Environments

As explored and verified in [48], the data collection for sound localization was carried out in a laboratory equipped with Wave-Field Synthesis (WFS) technology and an acoustically treated environment designed to faithfully replicate highly realistic real-world conditions. This laboratory, designed to minimize external interference and reflections from walls and ceilings, was equipped with an acoustically isolated system that ensured the accuracy of measurements.

**Figure 15:**
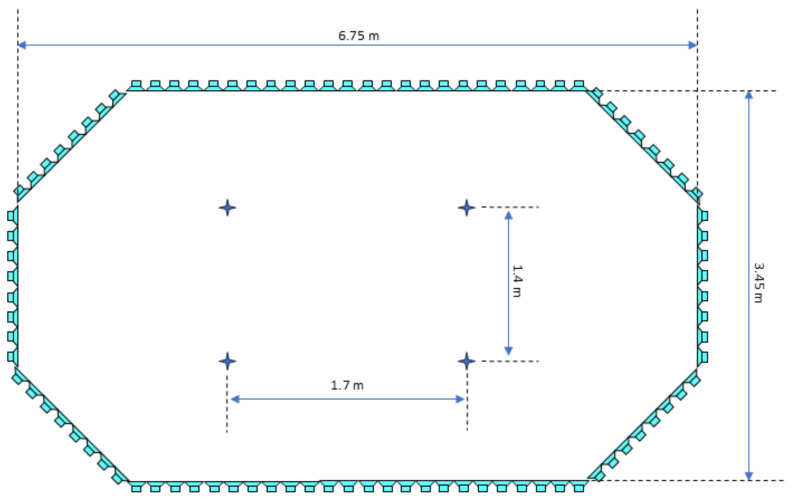
Experimental Setup in WFS for Semi-Synthetic Acoustic Field Data Recording. The Cross Star symbols represent the position of the microphones.

The audio system included a setup of 96 speakers arranged in an extended octagonal configuration, enabling the synthesis of realistic sound fields tailored to the available space. Regarding the microphones, the configuration shown in Figure 15 was followed, with dimensions of 1.7 m × 1.4 m, mimicking the microphone arrangement in a real vehicle. This setup facilitated the accurate capture of relevant signals, simulating the acoustic conditions of a moving vehicle from all directions. In order to obtain a frequency response that closely resembles real-world conditions, measurements were conducted with the microphones positioned within the ICECREAM structures.

**Figure 16:**
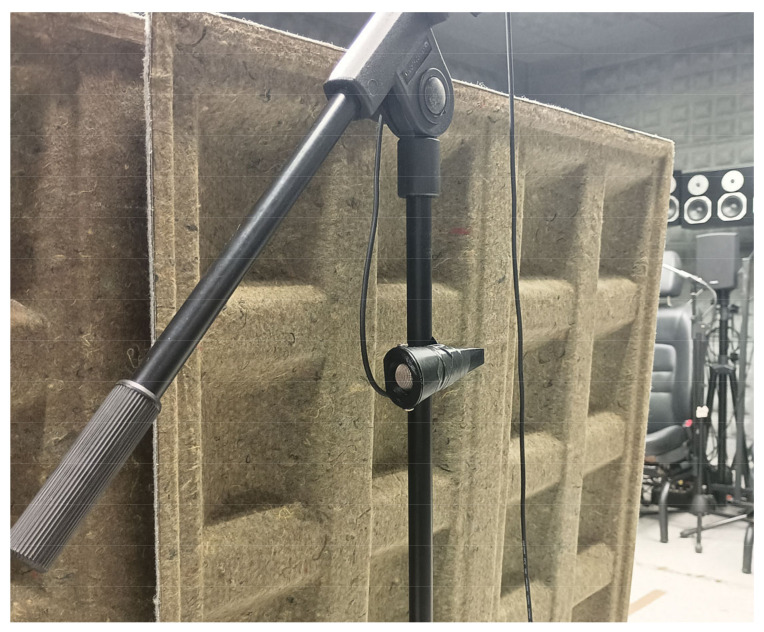
Experimental ICECREAM Setup in WFS for Semi-Synthetic Data Recording.

To enhance the emulation of a real-world environment, a structure composed of absorbent materials was implemented to replicate the acoustic effect produced by the vehicle’s body. This sound attenuation effect varies depending on the direction, as sound cannot pass through the vehicle’s structure. By placing these absorbent panels in front of the microphones, the sound incidence is modified in specific directions. This experimental arrangement is illustrated in Figure 16.

To emulate typical background noise in a vehicular environment, a road noise simulation was used, employing planar sound waves to ensure a homogeneous acoustic field distribution in all directions. This technique was crucial for realistically representing the sound environments, which was essential for the validity and reliability of the experiments.

The experimental procedure involved randomly selecting audio fragments from the A3CarScene database [50], as well as those recorded in Section 5.1, to replicate road noise. Additionally, the UrbanSound8K database [51] was used to obtain specific signals of interest, such as sirens and horns. During the experiment, road noise was simulated in a diffuse field, and sirens and horns were then emitted in specific directions, varying randomly in degree increments, covering a full circle in the azimuth plane. This approach allowed for a comprehensive evaluation of the system’s ability to detect and localize signals of interest in various spatial orientations, faithfully replicating real-world conditions.

The four microphones recorded the sound simultaneously, generating four audio channels for each pair of signals of interest and background noise. This process was repeated multiple times for all the samples from the UrbanSound8K dataset, obtaining the same samples with different background noise, resulting in more than 20,000 samples. To achieve this, an automated simulation system labeled each signal from the UrbanSound8K dataset with the corresponding fragment from the A3CarScene dataset or a previously recorded noise signal. Additionally, the system recorded the angle at which the signal of interest was incident during the recording. All recordings were sampled at 48,000 Hz.

### 5.3. Synthetic and Semi-Synthetic Outdoor Environment Generation

In the context of detecting and classifying close trajectories, it is essential to gather a large dataset of sounds originating from objects moving near a vehicle. To achieve this, a combination of data generated through computer simulations and those obtained from controlled laboratory environments was utilized to cover a broader range of potential scenarios.

Since sounds that persist over time during a trajectory are predominantly sirens, this part of the study focuses on such signals, employing the database presented in [52]. The simulation of moving sound is particularly challenging, as it depends on factors such as the varying speeds of the emitting object, which introduce a Doppler effect [53], as well as significant variability based on acoustic conditions in the environment. Consequently, the data collection strategy incorporated both laboratory simulations and the Wave-Field Synthesis (WFS) system.

For local computer simulations, the “pyroadacoustics” framework [54] was employed, enabling the emulation of sound from a moving object on a roadway while accounting for parameters such as air temperature, humidity, asphalt absorption, and speed, among others. This tool facilitated the generation of approximately 9500 audio files by replicating each element of the siren database [52] across various directions and speeds, covering the range of 10 km/h to 80 km/h, which corresponds to the typical speed range of emergency vehicles in urban environments.

One inherent limitation of using this system is that it emulates a free-field scenario, idealizing the acoustic environment without simulating the vehicle’s bodywork. This omission prevents the replication of the occlusion effects produced by the vehicle on microphones positioned opposite the sound trajectory. However, in real-world environments, microphones that are not obstructed by the vehicle do not experience occlusion, allowing valuable information to be obtained from these measurements.

To complement this dataset and specifically address this limitation, the setup described in Section 5.2 was employed to emulate vehicle occlusion within the laboratory environment. To ensure no single simulation method dominated the dataset, approximately 9500 samples were reproduced in the laboratory using the same information and siren database, but now simulating close trajectories to the vehicle with the Wave-Field Synthesis (WFS) system.

Finally, all samples were augmented with random noise segments extracted from the measurements detailed in Section 6, maintaining a signal-to-noise ratio (SNR) between −12 dB and 5 dB SPL. This approach enriched the dataset and provided a more realistic representation of acoustic conditions in scenarios involving close trajectories near the vehicle.

### 5.4. Motion and Real-World Environment Capture

Validation data acquisition is critical for objectively evaluating the performance of the developed models. Using the same samples for both training and validation introduces inherent bias, which could artificially inflate metrics such as precision and accuracy rates. To address this, new data were collected in real-world environments, allowing for the assessment of system performance under realistic operating conditions.

**Figure 17:**
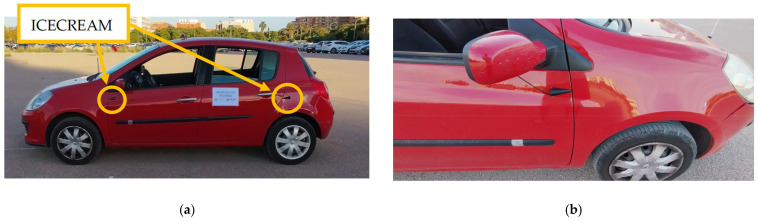
Vehicle Microphone Setup for Data Collection in Realistic Environments: (**a**) Lateral arrangement of microphone placement; (**b**) Enlarged view of microphone setup details.

The experimental setup for validation data collection involved installing four electret microphones equipped with ICECREAM structures on a vehicle, positioned at its four corners, as depicted in Figure 17. A Zoom H6 Handy Recorder was used to record the four audio channels, configured with a sampling rate of 48 kHz and 32-bit floating-point resolution. The microphone gain levels were standardized to ensure uniform signal representation across all channels, preventing any single signal from dominating the recordings and ensuring consistency in the measurements.

The selected testing field was an open-air parking lot, where measurements were conducted under two primary configurations: static sirens and moving sirens. The parking lot featured an asphalt surface, enhancing the validity of the results for real-world environments, as asphalt is the typical surface in urban and interurban roads.

**Figure 18:**
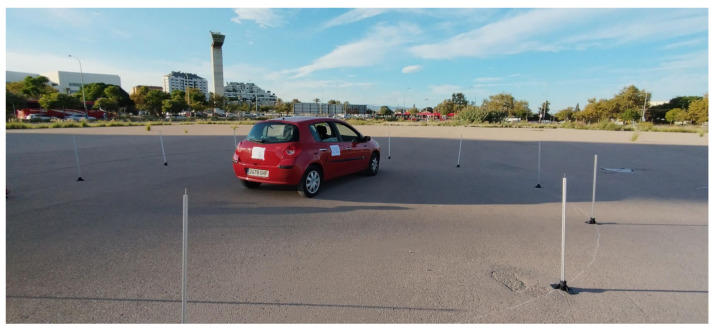
Static Siren Data Collection Experimental Setup. The bars indicate the sound emission points, spaced 30° apart.

For the collection of static data, a circle was drawn on the ground around the vehicle, with markers placed every 30°, starting at 0° and extending to 330°, as illustrated in Figure 18. This angular resolution was deemed sufficient to represent a practical scenario in which a driver can accurately localize the sound source in space. Multiple concentric circles with radii ranging from 5 to 50 m were established, enabling an evaluation of the impact of distance on the accuracy of sound source localization.

To emulate the sound of a siren, a TOA ER-520S megaphone with a built-in siren signal was used, created by TOA Electronics, a company founded in 1934 in Kobe, Japan, and a leader in public address systems. During the experiment, the siren was activated for 10 s at each point along the circumference.

**Figure 19:**
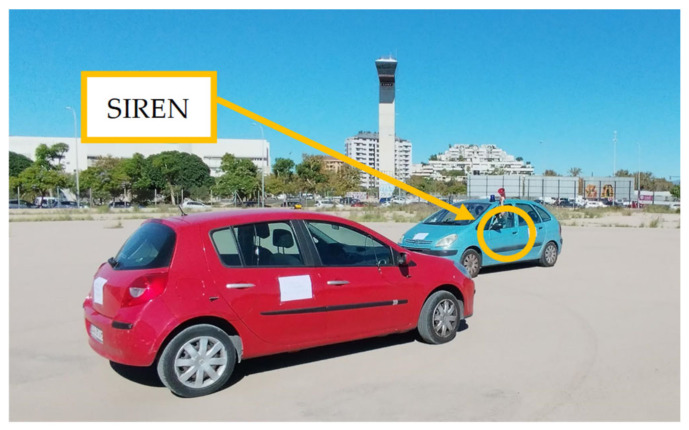
Set-up of the close trajectory experiment.

For the dynamic data collection, the microphone setup on the primary vehicle remained unchanged. However, the megaphone was mounted on a second vehicle in motion. This secondary vehicle simulated close-proximity trajectories around the primary stationary vehicle, as depicted in Figure 19. This approach allowed for the acquisition of data from moving sirens in close environments, complementing the previously collected static data.

The primary objective of this experiment was to capture data representing close-range trajectories relative to the stationary vehicle, simulating dynamic scenarios as described in Section 4.3. This design was critical for analyzing the system’s response under non-static conditions, where both the sound source and its localization continuously change.

Given that the system’s analysis operates using 1-s sliding windows with a total buffer of 4 s, close-proximity movement can introduce significant variations within the analysis window. To address these challenges, the microphone setup remained mounted on the stationary vehicle, while the megaphone was carried by the moving vehicle along the predefined close-proximity trajectories shown in Figure 19. This methodology ensured the collection of representative data from all possible trajectory types, thereby enhancing the robustness of subsequent model validation.

To facilitate the labeling and analysis of data after the experiment, an emergency light synchronized with video recordings was utilized. This setup allowed for precise identification of the moments when close-proximity trajectories occurred and their specific paths. This method provided a clear reference point, ensuring accurate validation of the data collected in dynamic environments.

Finally, additional measurements were taken by aligning the megaphone along a straight trajectory perpendicular to the vehicle. In this configuration, 5-s sound recordings were made at various distances, ranging from 5 m to 110 m, allowing the analysis of how distance affects the quality of the received signal.

This validation dataset, obtained under real-world conditions and diversified across static and dynamic configurations, provides a robust foundation for evaluating the system’s performance, ensuring its reliability and accuracy in real-world scenarios.

## 6. Results and Validation

This section presents a comprehensive evaluation of our integrated system, demonstrating how careful hardware design (Section 3), sophisticated AI architecture (Section 4), and robust training methodology (Section 5) combine to create a highly effective emergency sound detection and localization system. The results obtained during both the training and validation phases of the system’s components are analyzed, evaluating the performance of the implemented AI models and classical algorithms. Key metrics related to the system’s overall performance, including its ability to accurately detect and classify sounds in noisy and dynamic environments, are highlighted.

All AI training processes followed a standardized data partitioning approach, using 80% of the data for training, 10% for validation, and 10% for testing, ensuring robust model evaluation and minimizing the risk of overfitting to the training dataset. The validation process employs the diverse dataset described in Section 5, with particular emphasis on challenging real-world operational conditions.

### 6.1. ICECREAM Results

**Figure 20:**
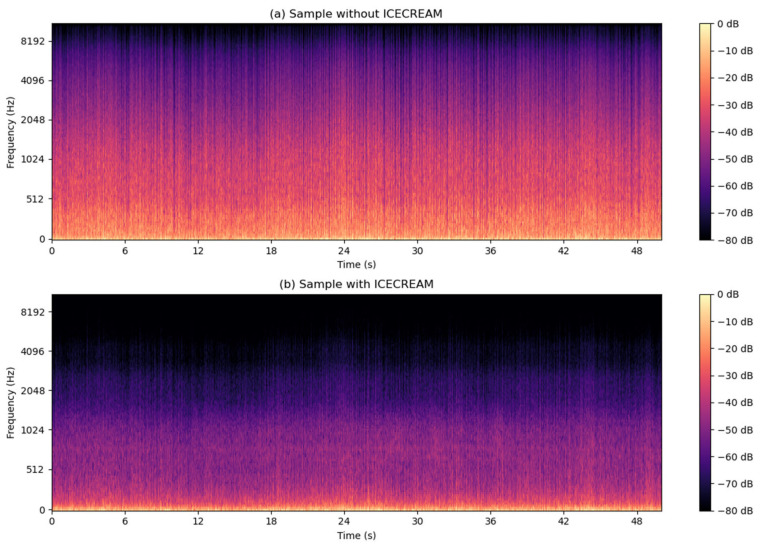
Spectrogram of real road environment noise with the car in motion: (**a**) exterior microphone without ICECREAM; (**b**) exterior microphone with ICECREAM.

As demonstrated in [33], the windproof structure achieves a significant reduction in wind noise, as shown in Figure 20. This configuration has proven effective both in field tests and in specialized laboratory settings, enabling the capture of high-quality audio even under real driving conditions.

**Figure 21:**
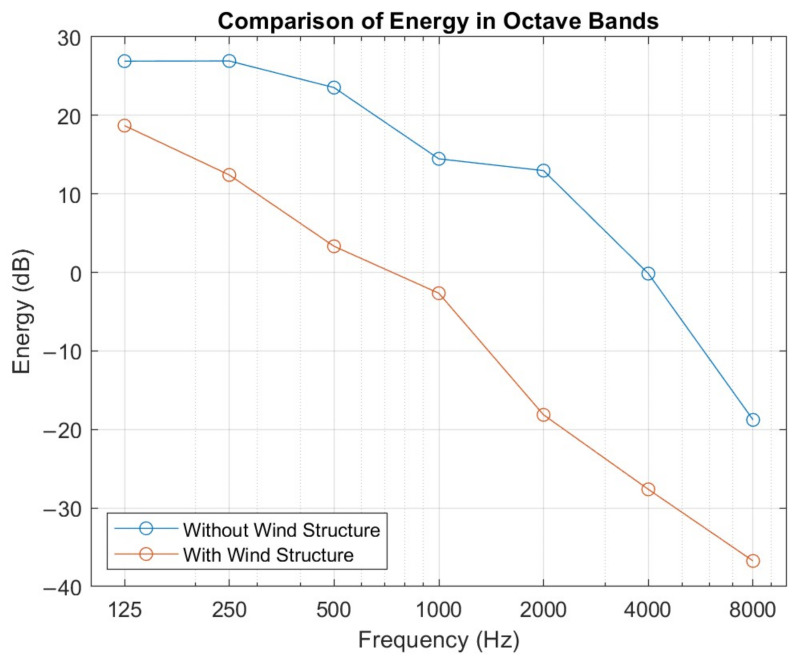
Comparison of energy in octave bands of real road environment noise with the car in motion, showing the difference between the noise with the wind protection structure (red line) and without the wind protection structure (blue line).

Figure 21 more accurately illustrates the attenuation of noise in octave bands, highlighting a significant reduction in energy levels across all bands when using the protective structure. Due to the absorption of the materials that fill the structure and the nature of the sound, the noise reduction is much more pronounced in higher frequencies than in lower frequencies, with a noise reduction exceeding 25 dB. This high-frequency attenuation helps minimize interference in the frequency ranges of emergency sounds, such as sirens, ensuring a clear and stable signal. The residual low-frequency noise will be addressed later in Section 6.3.

The implementation of this aerodynamic protective structure has enabled the system to capture high-quality audio even in real driving conditions at speeds of up to 120 km/h. This protection against wind and vibrations is an essential component within the sound detection and localization system, as it ensures that the AI algorithms receive a clear, interference-free signal, thereby enhancing the accuracy of emergency sound localization and recognition.

### 6.2. AI-Based Detection System Results

#### 6.2.1. Train Results

The emergency sound detection model is based on advanced transformer architectures, widely recognized for their ability to identify complex relationships in sequential data. However, these networks require high computational capacity due to the intensive nature of their processing and the large volumes of data they handle. This challenge is exacerbated when using extensive datasets such as AudioSet [55], which contains over 2.2 million audio samples.

To address this issue, a transfer learning approach was employed [56], leveraging a pre-trained model on the AudioSet dataset across 527 classes developed by MIT [57]. This pre-trained model provides a solid foundation of general acoustic representations, allowing the training to focus on specific tasks without requiring the full adjustment of all model parameters.

During the fine-tuning stage, the weights of the multi-attention layers of the AST were frozen, and the final classification layers of the model were retrained using the UrbanSound8K dataset, which includes labeled samples of sirens and horns. To simplify the classification, the other classes present in UrbanSound8K were grouped under the “nothing” category. This fine-tuning process was carried out using 10-fold cross-validation, a widely used technique for evaluating model robustness and preventing overfitting, as recommended in [51].

This approach, which combines a pre-trained model with specific fine-tuning techniques, significantly reduced training times and computational resources while maintaining high classification accuracy.

Additionally, adaptive learning rate strategies were implemented to optimize model convergence during training, as well as early stopping techniques to prevent overfitting. The model was trained using a cross-entropy loss function [58] and the ADAM optimizer [59]. The ADAM optimizer was chosen for its ability to adaptively adjust learning rates for each parameter during training. This dynamic adjustment is particularly beneficial for complex models like the Audio Spectrogram Transformer (AST), which operates on large-scale audio spectrograms and contains many parameters. By incorporating both momentum and adaptive learning rates, ADAM enhances the efficiency of training, enabling better convergence and faster optimization compared to traditional optimizers such as SGD (Stochastic Gradient Descent).

The performance of the model during training was evaluated using standard classification metrics such as accuracy, recall, and F1-Score. These metrics provide a comprehensive view of the model’s performance, particularly in complex scenarios or with imbalanced classes, where it is important to evaluate not only how many predictions are correct but also how relevant those predictions are.

Precision, defined by Equation (3), measures the proportion of positive predictions made by the model that are correct. It is a key metric for determining the model’s ability to avoid false positives, which is critical in this case, where incorrectly identifying a non-emergency sound as an emergency can be problematic.

(3) $$Precision\;=\;\frac{True\;Positives}{True\;Positives\;+\;False\;Positives},$$

On the other hand, recall, as described in Equation (4), evaluates the proportion of actual positive events that the model correctly identified. This is especially important in critical systems, as missing an event such as a siren could lead to a significant failure.

(4) $$Recall\;=\;\frac{True\;Positives}{True\;Positives\;+\;False\;Negatives},$$

The F1-Score, defined in Equation (5), combines precision and recall into a single metric through their harmonic mean, providing a balanced analysis of the model’s performance. It is particularly useful for evaluating systems where a balance between minimizing false positives and false negatives is desired.

(5) $$F1\text{-}Score\;=\;\frac{2\;\cdot \;Precision\;\cdot \;Recall}{Precision\;+\;Recall},$$

Accuracy, as defined by Equation (6), measures the overall proportion of correct predictions made by the model, including both true positives and true negatives. It is a fundamental metric in evaluating model performance, providing a clear indication of how often the model is correct across all classifications. However, while accuracy is useful, it may not fully capture the performance in imbalanced datasets, where one class may dominate the other.

(6) $$Accuracy\;=\;\frac{True\;Positives\;+\;True\;Negatives}{Total\;Predictions},$$

The results detailed in Table 1 reveal an F1-Score close to 96%, indicating an outstanding balance between precision and recall. These values, along with equally high accuracy, confirm that the model can consistently and accurately identify emergency events, even in adverse acoustic conditions. The high accuracy achieved reinforces the system’s reliability in classifying both relevant and irrelevant sounds, demonstrating its suitability for critical applications in real-world environments.

#### 6.2.2. Validation Results

For this analysis, the audio obtained from the measurements described in Section 5 was manually labeled, indicating whether or not a siren was present in the signal at one-second intervals. Subsequently, the results generated by the detector were compared with the actual labels, allowing for an accurate evaluation of the system’s performance.

The model achieved the following results: Recall: 100%, F1-Score: 96%, and Precision: 92%. These values reflect an exceptional ability to detect sirens, with precise identification of emergency events, minimizing false negatives while maintaining a low level of false positives.

**Figure 22:**
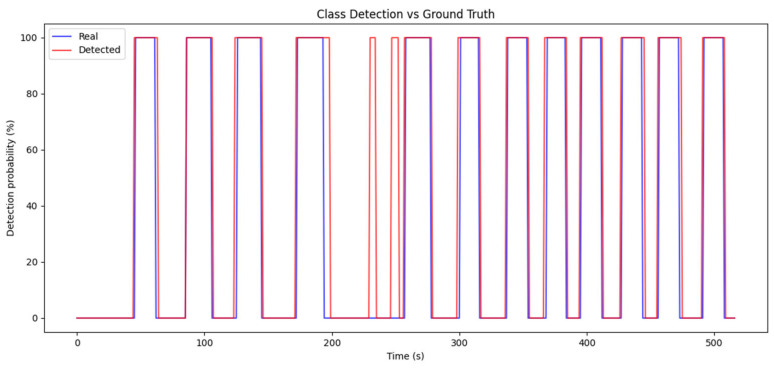
Comparison between model predictions and the actual ground truth, with results obtained every second using a sliding window technique.

Figure 22 shows a comparison between the detections made by the model and the ground truth. As can be seen, the detection is nearly perfect, which is reflected in the high recall value, as the model does not miss any siren detection. However, it is also noticeable that when working with sliding windows of 4 s, a remnant of the siren may still appear in the buffer even after it has ended, which causes a slight reduction in precision compared to the actual values. This phenomenon would not represent a significant problem in practice. Some false positives are also observed, contributing to a slight decrease in precision. This issue could be addressed by implementing a confidence system that compares detection results with previous and subsequent ones to determine whether the emergency sound is relevant or not.

In addition, the high agreement between the model’s predictions and the actual labels demonstrates that the detector can reduce both false positives and false negatives. This ensures that the system not only identifies emergency events effectively but also avoids incorrect activations, maintaining a high level of accuracy and reliability. Despite some false positives and the slight reduction in precision due to the use of the sliding window, the overall performance remains highly satisfactory and suitable for implementation in real-world conditions.

For horn sounds, the ESC-50 dataset [60] was used as a reference for the analysis. Although ideally, direct measurements of horns in various spatial positions would have been conducted, technical difficulties arose during the experimental design. Specifically, accurately measuring the angular incidence of horn sound in a stationary vehicle proved to be a challenge due to the irregular dispersion of sound and variations in its propagation. These limitations compromised the accuracy of the measurements and, therefore, did not allow for a reliable representation to validate the model under these conditions.

The use of the ESC-50 dataset, while different from on-site measurements, provided a reliable set of labeled data that allowed for the evaluation of the model’s response to horn sound events in simulated scenarios, resulting in a correct identification rate of 97.5%.

### 6.3. AI-Based Cleaning System Results

#### 6.3.1. Train Results

This section details the software component of the audio cleaning system, which complements the hardware section presented previously in Section 3 and Section 4.2. In this phase, the results obtained from training an artificial intelligence model using the data described in Section 5 are presented. For constructing this dataset, clean audio signals were extracted from the UrbanSound8K and ESC-50 datasets, to which ambient noise from recordings made with the ICECREAM system, described in Section 5.2, was added. This combination of clean and noisy signals constitutes the dataset used to train the model, with spectrograms of the noisy signals as inputs and the spectrograms of the clean signals as outputs.

The model was trained using advanced techniques such as early stopping and adaptive learning rate, implementing the ADAM optimizer. During the training, evaluation metrics such as signal-to-noise ratio (SI-SNR) and signal-to-distortion ratio (SI-SDR) were employed, which allowed quantifying the noise reduction and distortion of the signal. These metrics provide an accurate measure of the model’s quality, evaluating both the preservation of the original signal’s quality and the effectiveness of the cleaning applied. Quantitatively, SI-SNR values significantly improved, rising from −12.59 dB to 8.62 dB, while SI-SDR values increased from −0.96 dB to 11.65 dB. These changes reflect a notable improvement in the signal-to-noise ratio, as well as a significant reduction in distortion of the processed signals, indicating that the model has successfully learned to separate the emergency signal from the ambient noise.

#### 6.3.2. Validation Results

**Figure 23:**
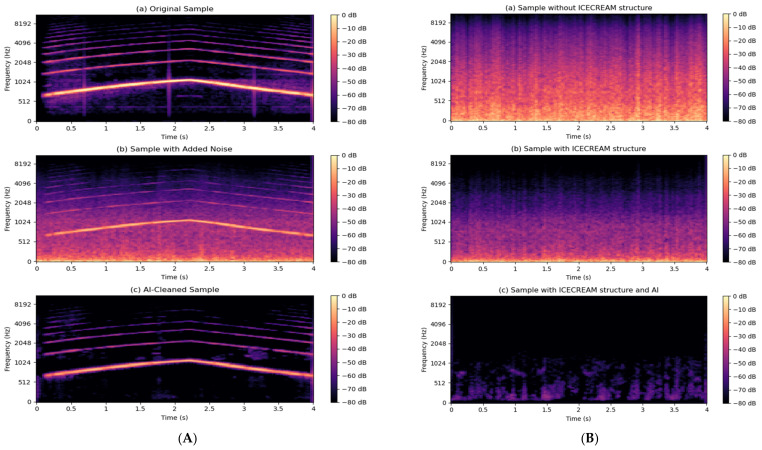
The Comparative Operation of the Cleaning Process: (**A**) AI-based cleaning system only; (**B**) Comparison of real-world environment noise-cleaning process using both ICECREAM and AI-based cleaning.

Figure 23 illustrates a comparative example of a sample before and after processing by the model, highlighting the visual improvement in the spectrogram. As observed, the cleaning process significantly reduces interference while maintaining the integrity of the emergency signal features. This validates the effectiveness of the system for real-world scenarios with signals contaminated by noise.

Additionally, in Figure 23A, it is observed that the final sample, after completing the cleaning process, contains even less noise than the original signal recorded in databases such as UrbanSound8K or ESC-50. This phenomenon occurs because these databases contain samples with controlled levels of background noise designed to promote the generalization of artificial intelligence models across various training scenarios. This allows AI systems to become more robust against contaminated signals, but when the cleaning process is applied, the model is able to remove both the added noise and some of the inherent noise in the base signal, achieving a higher level of acoustic purity. This result reinforces the effectiveness of the system, not only in reducing noise but also in its ability to enhance the overall quality of the processed signals.

In addition to the previously described results for a siren sample, a detailed comparison of the complete cleaning process is provided. This starts with the effect of the windproof structure and continues with the joint action of this structure and the artificial intelligence model. For this evaluation, a sample from Section 5.1 was used, which corresponds to a recording where only environmental noise data, including wind and tire roll, were collected, with no emergency signals present.

The image presented in Figure 23B illustrates how the system significantly reduces noise in two key stages: first, with the attenuation provided by the windproof structure, and second, with the additional refinement carried out by the AI model. As seen, the residual noise is drastically minimized, which demonstrates the effectiveness of combining both strategies. This analysis not only validates the system’s ability to operate in noisy environments but also highlights the importance of integrating physical solutions and advanced algorithms to achieve optimal acoustic cleaning levels.

### 6.4. AI-Based Static Localization System Results

#### 6.4.1. Train Results

To address the problem of static localization, the data obtained from the collection described in Section 5.2 were used, providing a dataset of over 18.000 samples for training the neural network. This training was carried out using techniques such as early stopping and adaptive learning rate, which allowed for optimizing the model’s convergence and preventing overfitting issues. Additionally, the ADAM optimizer was employed to efficiently manage the weight updates.

Since the problem to be solved is a circular regression, where values of 359° and 0° are adjacent, a standard loss function could not be applied. In this context, using a linear metric would have introduced significant errors at the boundaries of the circle. Therefore, a specific loss function, called Circular Cosine Loss, was designed, which takes into account the circular nature of the data. This function is based on the normalized angular difference and its relationship with the cosine, as defined in the following equation:

Circular Cosine Loss = ∑(1 − cos(Δθ)),(7)

where Δθ is defined as the angular difference between the model predictions and the actual values, normalized to the range of a full circle [0, 2π).

This loss function allows small angular differences to be assigned minimal errors, while maximum errors, equivalent to 180°, receive a higher penalty. During training, the model continuously recorded the mean error in degrees to monitor its performance. At the end of the training, a mean error of 4.39° with a standard deviation of 8.7° was achieved, demonstrating that the model is capable of locating sounds with high precision under validation conditions. These values reflect robust performance, validating the effectiveness of the methodology applied to solve the static localization problem in real-world environments.

#### 6.4.2. Validation Results

Two main validations were conducted to assess the performance of the localization method: one in the laboratory using the WFS system described in Section 5.2 and another in a real-world environment, as outlined in Section 5.4. The difference in the number of samples between the two cases is due to the fact that generating simulated samples in a controlled environment is significantly easier than collecting them experimentally, considering the time and resource limitations associated with field measurements.

For the simulated data in the laboratory, the same setup and procedure described in Section 5.2 were used. However, this time, the ESC-50 database, which includes emergency sound recordings, was employed. More than 4600 simulated samples were generated, with sources positioned in increments of 30° in the azimuthal plane. This level of resolution is considered sufficient to accurately identify the direction of sounds on roads, providing the driver with clear and useful information. During the analysis, a correct prediction was defined as any estimation whose angular estimate fell within a ±15° margin from the actual angle, ensuring that the final localization would be correct for practical use.

The system achieved an accuracy of 94.29% on these simulated samples, validating its ability to identify emergency sounds in controlled environments. Additionally, the mean angular error and its standard deviation were calculated, yielding a mean error of 5.12° and a standard deviation of 9.85°, demonstrating highly consistent performance suitable for real-world applications. These results confirm the effectiveness of the method under controlled conditions, providing a basis for its evaluation in real-world environments. Furthermore, they represent a significant improvement over many of the studies presented in [7], which rely on simulated data, highlighting the advancements made by the proposed system.

**Figure 24:**
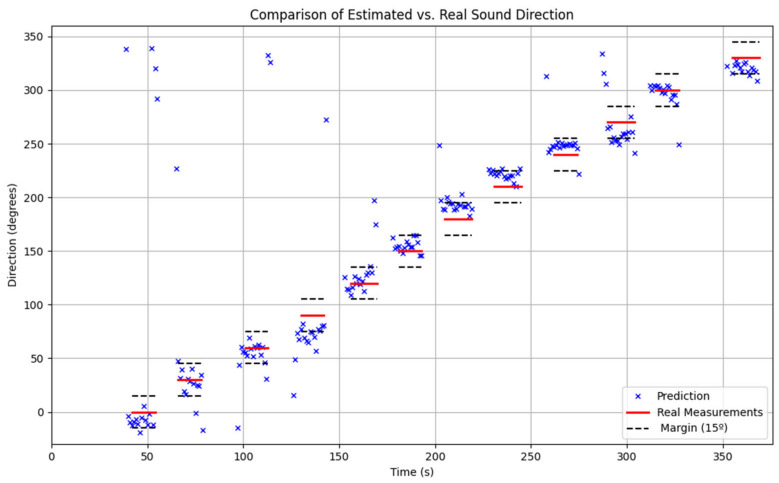
Comparison between Predictions and Ground Truth of Real-World Measurements, with results obtained every second using a sliding window technique.

For the real-world validation, a comparison was made between the samples obtained from the static localization system with sliding window and the actual values. The obtained results are presented in Figure 24.

This figure illustrates a strong correlation between the actual values and those estimated by the system. Although a slight dispersion from the true values is identified, all predictions remain within the ±15° margin, ensuring sufficient accuracy for practical applications.

While the correlation between the real and detected values is high, some outliers are present. These outliers may arise when a portion of the signal falls within the window being analyzed during the sliding window process, potentially causing minor anomalies in the predictions. This issue can be mitigated in practical applications through the implementation of a confidence system. Such a system would compare current predictions with the values from the previous and subsequent seconds, discarding any values that fall outside the acceptable range and using the predictions from the following second to maintain the system’s accuracy.

After a thorough analysis of all the samples collected in real-world environments, an average error of 10.30° and a standard deviation of 8.55° were determined. These results confirm the system’s ability to operate with high reliability in uncontrolled acoustic conditions, improving upon the results of the most current and comparable state-of-the-art study [28], which exclusively examined real-world measurements. They validate the effectiveness of the implemented approach for static localization in real-world situations.

### 6.5. AI-Based Close-Passing Trajectory System Results

#### 6.5.1. Train Results

As explained in Section 4.3, the first step involves training the model using the data obtained in Section 5.3. For this training, early stopping techniques are employed along with an adaptive learning rate, the ADAM optimizer, and a cross-entropy loss function, in order to classify the different nearby trajectories around the vehicle. These trajectories correspond to the 8 labels of the various positions along the trajectory, as shown in Section 4.3.

**Figure 25:**
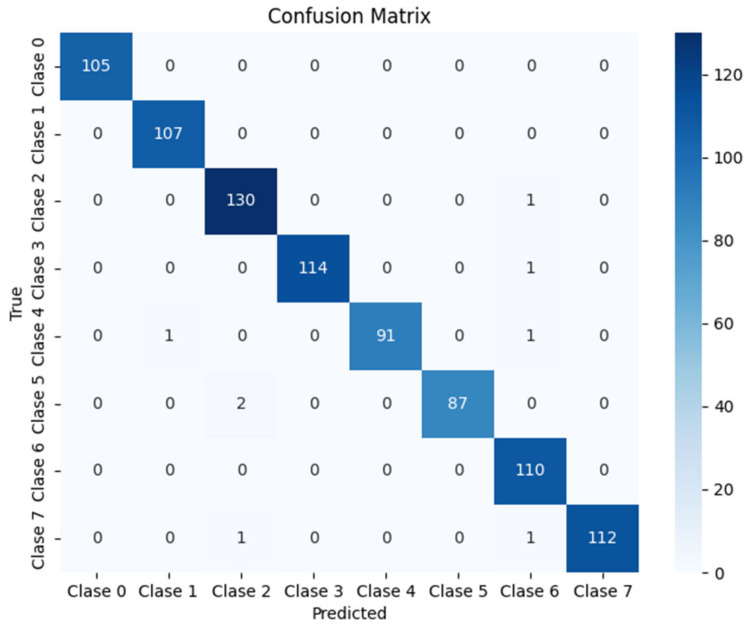
Confusion Matrix of the AI-based Close-Passing Trajectory System Training with Different Types of Close Trajectories.

This training process adjusts both the convolutional layers of the ResNet-18 and the fully connected layers, which are responsible for transforming the pattern recognition knowledge learned by the convolutional layers into the classification of the different nearby trajectories. After training the model, an accuracy of 99.07% was achieved on the test dataset, as shown in Figure 25.

Once the classification model for identifying close trajectories is trained, the weights of the convolutional layers in the ResNet are frozen to prevent them from being adjusted in the next process. Then, a new detection branch is added and trained, with the goal of determining the presence of a close-passing event using the data described in Section 4.3, which include samples both with and without close movement. For this training, early stopping techniques, adaptive learning rate, the ADAM optimizer, and a binary cross-entropy loss function were once again employed, as there are only two classes: “activated” (Class 1) or “not activated” (Class 0).

**Figure 26:**
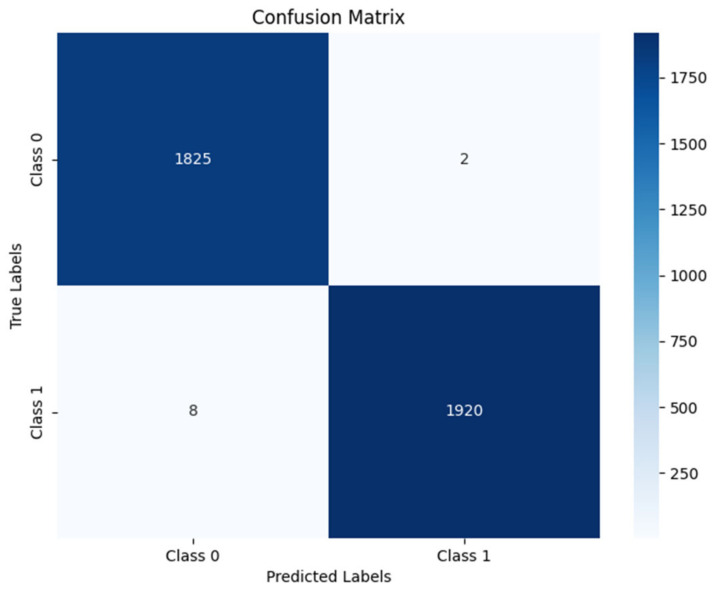
Confusion matrix of the Close-Passing Trajectory Detection branch training.

At the end of the training, an accuracy and F1-Score of almost 100% were achieved, resulting in a confusion matrix, as shown in Figure 26. The results demonstrate that the detection on the test set is nearly perfect, highlighting the effectiveness and high reliability of the model in accurately and robustly identifying close-passing trajectories in real-world scenarios.

Upon completing this process, a fully integrated model is obtained, incorporating both branches: one for detecting the presence of a close-passing trajectory and another for classifying the type of trajectory. This approach allows the system to not only identify the presence of a close-passing trajectory but also accurately characterize the type of event occurring around the vehicle.

#### 6.5.2. Validation Results

To validate the results obtained, the samples collected in Section 5.4 in real-world environments were analyzed. These samples consist of 31 measurements, a limited number due to the complexity of recording such sounds in real conditions, as it requires the collaboration of multiple vehicles and an appropriate space where they can move without compromising road safety.

**Figure 27:**
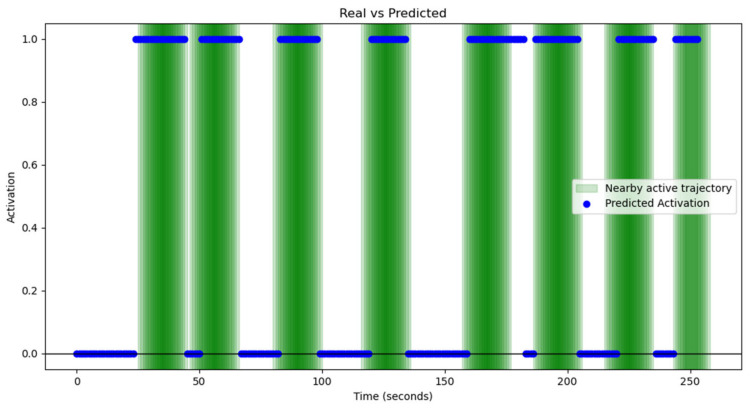
Comparison between Predictions and Ground Truth of Real-World Experiment, with results obtained every second using a sliding window technique.

First, the system’s performance in detecting close-passing trajectories in real-world environments was evaluated. The results showed a precision of 87.88%, a recall of 77.33%, and an F1-Score of 82.27%, reflecting satisfactory model performance. Figure 27 presents a representation of the temporal activations generated by the model compared to the green bands, which indicate the actual moments when close-passing trajectories occur. As the metrics suggest, the system’s predictions largely align with the real events’ timings.

It is important to highlight that since there is no prior automated system for determining the intervals of close-passing trajectories, the annotation of these moments was performed manually by combining the acoustic information from the audio files with the analysis of videos recorded during the experiments. Therefore, the ground truth may contain slight inaccuracies due to the limitations inherent in this manual procedure.

Additionally, since the system uses a sliding window analysis of 4 s, it is possible that remnants of the close-passing trajectory remain in the analysis buffer even after the event has ended, leading to residual positive activations. While their impact on practical applications would be minimal, this issue could be mitigated by implementing confidence algorithms that take into account the results of previous and subsequent predictions, ensuring greater robustness in detection.

**Figure 28:**
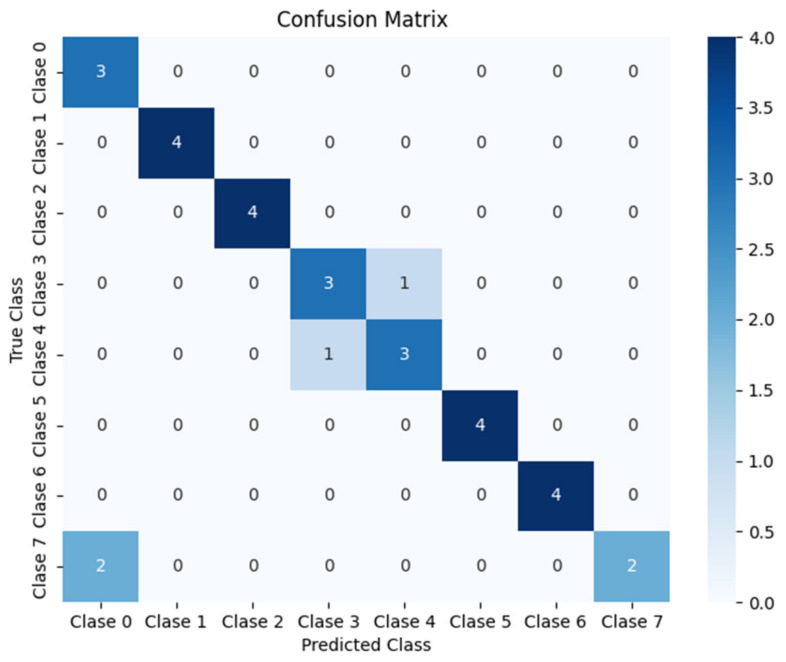
Confusion matrix of the Close-Passing Trajectory Classification branch training.

Once the detection phase was validated, the classification of close trajectories was evaluated. For this purpose, 4-s clips covering the entire close trajectory were selected, and the system was allowed to perform the classification. The model achieved an accuracy of 87.1% during this phase, and the corresponding confusion matrix is presented in Figure 28.

In summary, the results indicate that while the detection of close-passing trajectories is effective, there is room for improvement, particularly in terms of recall. This may be related to experimental factors and the inherent challenges of data collection in real-world environments. On the other hand, the classification of close-passing trajectories demonstrated outstanding performance, reflecting the model’s high ability to discriminate between different trajectories in real-world situations.

### 6.6. Distance Estimation Algorithm Results

The final evaluated block corresponds to the distance classification described earlier in Section 4.5. This block does not require training, as it is based on the direct analysis of the samples obtained in Section 5.4, where each audio file is associated with its corresponding distance. The classification employs harmonic energy ratios and defined thresholds to determine the proximity of the sound source.

After extensive practical experimentation and analysis, specific thresholds were determined for harmonic energy ratios to classify distance accurately. For the near range (0–20 m), the threshold for the energy ratio between the sixth and third harmonics is set at 70. If the value is below 70, the sound is classified as near. Conversely, for the far range (greater than 50 m), the threshold for the energy ratio between fundamental frequency energy and the third harmonic energy is set at 13. If this value exceeds 13, the sound is classified as far.

**Figure 29:**
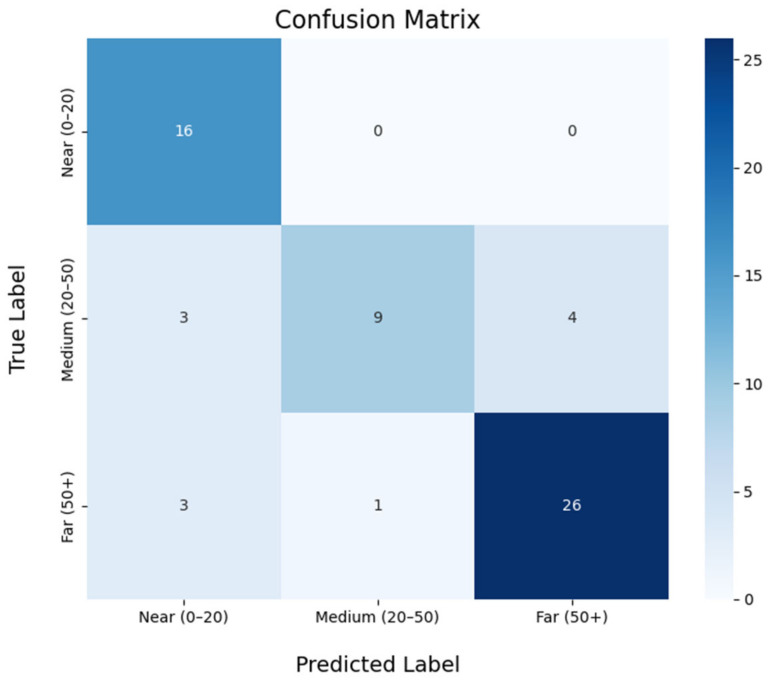
Confusion matrix of validation results for the distance estimation algorithm.

After applying the harmonic comparison algorithm and these defined thresholds, the system achieved an accuracy of 82.26%, with the confusion matrix shown in Figure 29. The defined distance ranges—close (0–20 m), medium (20–50 m), and far (greater than 50 m)—are considered optimal as they provide the driver with a clear and actionable interpretation of the emergency vehicle’s proximity. This enables fast and effective decision-making to enhance road safety.

Additionally, as this process runs continuously at one-second intervals, the system provides real-time updates on the distance of the emergency sound. This allows not only the classification of the current distance of the emergency vehicle but also the analysis of its trend—whether the sound is getting closer or farther from the vehicle. This analysis is further enhanced by evaluating the waveform amplitude from the first sound detection to the current moment, offering a clear and dynamic representation of the sound’s behavior in both space and time.

Looking ahead, it is expected that this verification can be refined with more samples, as the current dataset may not be large enough to yield statistically significant conclusions. It can also be observed that the classification in the medium-distance range is less effective compared to the close and far ranges. This algorithm represents a first approach to solving the problem of real-time distance estimation of an emergency sound source in open-field environments. Future developments, including expanded datasets and potential integration of AI-based methods, would enhance the system’s robustness and accuracy.

## 7. Conclusions

This work successfully demonstrates the design, implementation, and validation of an advanced Advanced Driver Assistance System (ADAS) for the detection and localization of emergency sounds, such as sirens and horns, in automotive environments. The originality of the proposed system lies in its comprehensive approach, combining groundbreaking hardware and software innovations to address the challenges posed by dynamic, noisy, and real-world road conditions. This comprehensive approach significantly outperforms previous methods discussed in the introduction, offering a more robust and reliable solution for emergency sound detection and localization in challenging acoustic environments. Central to this innovation is the development of the aerodynamic structure, termed ICECREAM, which significantly advances acoustic hardware design for vehicular applications. By mitigating wind noise and microphone vibrations, the structure achieves a noise energy reduction of over 25 dB in central frequencies, enabling high-quality audio capture even at vehicle speeds of up to 120 km/h. This hardware advancement is further complemented by an AI-driven audio cleaning system, which achieves remarkable improvements in audio clarity, including a 21.21 dB increase in SI-SNR and a 12.61 dB improvement in SI-SDR, setting new benchmarks in the field of emergency sound processing.

On the software side, the system integrates state-of-the-art AI architectures, including transformer-based models for emergency sound detection and convolutional neural networks (CNNs) such as ResNet-18 and U-Net for sound localization and noise reduction. The adoption of a sliding window framework ensures real-time analysis of overlapping four-second audio segments, continuously providing drivers with up-to-date information on the direction, proximity, and trajectory of emergency sounds. This capability is further enhanced by the system’s real-time visualization module, which intuitively conveys critical situational information to the driver.

The experimental validation of the system demonstrates its effectiveness and reliability across diverse scenarios. Detection accuracy reached 98.86% with synthetic data and 97.5% with real-world measurements, while localization performance achieved an average angular error of 5.12° in simulations and 10.30° in real-world conditions, with an overall localization accuracy of 94.29% within ±15° margins. Additionally, the system exhibits robust performance in detecting close-passing trajectory events, with a precision of 87.88%, recall of 77.33%, and F1-Score of 82.27%. These results highlight the system’s potential to improve driver response times and situational awareness, offering a reliable and practical solution for integration into commercial ADAS technologies to enhance road safety.

This research also makes significant contributions to the field by addressing critical gaps in current systems. Unlike many existing approaches that focus exclusively on hardware or software, this work bridges the two domains to deliver a comprehensive solution. The hybrid AI architecture, combining transformers and CNNs, achieves superior performance compared to prior methods, particularly in the complex acoustic conditions of vehicular environments. Furthermore, the inclusion of a real-time visualization system addresses a key limitation in current ADAS technologies by providing actionable information to drivers in emergency situations.

While the system demonstrates high performance, there remain opportunities for further improvement. Expanding the dataset with more diverse and representative samples could enhance the accuracy of close-passing trajectory detection. Additionally, the integration of acoustic data with multimodal systems, incorporating visual sensors, could further optimize performance by leveraging complementary data streams. Such fusion would be particularly beneficial for scenarios involving approaching emergency vehicles, offering greater robustness and precision in complex and dynamic environments.

Future research will focus on integrating the system into vehicle onboard platforms to evaluate its performance in real-world driving scenarios. This step will require addressing challenges such as compliance with stringent safety regulations and ensuring that the system operates symbiotically with existing vehicle components. Collaboration with automotive manufacturers will be essential to design solutions that meet these requirements while maintaining the system’s safety and efficiency. These challenges represent an opportunity to develop innovative strategies that further advance acoustic-based ADAS technologies, maximizing their impact on road safety and emergency response systems.

This work not only advances the state-of-the-art in emergency sound detection and localization but also establishes a solid foundation for future developments in ADAS technologies. By introducing novel hardware and software solutions validated through rigorous experimental testing, this study provides a significant contribution to both academic knowledge and industrial practice in the field of automotive safety systems.

## 8. Patents

The technology/results/methods described in this paper are being protected through a patent application currently under consideration.

## Figures and Tables

**Table 1:** Results of the detection train.

K-Fold	Accuracy (%)	Precision (%)	Recall (%)	F1-Score (%)
1	99.89	99.43	100.00	99.71
2	98.99	99.43	93.04	96.10
3	98.92	97.97	96.32	97.13
4	98.18	95.56	90.98	92.79
5	99.15	99.31	96.43	97.80
6	98.66	95.91	93.92	94.70
7	98.09	89.95	91.23	90.52
8	99.13	98.70	96.88	97.78
9	99.88	100.00	98.44	99.21
10	97.72	96.38	89.14	92.56
Mean	98.86	97.26	94.64	95.93

## Data Availability

Data are contained within the article.
